# Bilateral Myelomatous Pleural Effusion in a Patient with IgA Kappa Multiple Myeloma

**DOI:** 10.7759/cureus.1238

**Published:** 2017-05-10

**Authors:** Rebecca Asiamah, Shiva Kumar Mukkamalla, Tanmay Sahai, Xiao Ping Zhou, Eric Han, Vincent Armenio

**Affiliations:** 1 Internal Medicine, Roger Williams Medical Center; 2 Internal Medicine/Hematology and Oncology, Roger Williams Medical Center; 3 Pathology, Roger Williams Medical Center

**Keywords:** multiple myeloma, pleural effusion, bad prognosis

## Abstract

Multiple myelomas is a neoplastic plasma cell disorder that accounts for one percent of all cancers and 13% of hematologic malignancies. Although primarily known to be a bone marrow disorder, it can metastasize to extramedullary sites or it can present as a solitary extramedullary plasmacytoma. Primary pleural effusion from myeloma is rare, occurring in less than one percent of the patients. The following case report highlights a case of bilateral pleural effusion, directly attributable to multiple myeloma after other causes were ruled out. The diagnosis was made using cytology and immunohistochemical (IHC) staining of the pleural fluid. Myelomatous pleural effusion (MPE) is a poor prognostic feature heralding an aggressive underlying disease state, as represented in this case.

## Introduction

Multiple myelomas is a neoplastic plasma cell disorder that is characterized by clonal proliferation of malignant plasma cells [1]. It accounts for one percent of all cancers in the United States with an incidence of about four to five cases per 100,000 population, as estimated in 2016 [2]. Although primarily known to be a bone marrow disorder, it can metastasize to extramedullary sites or can be present as a solitary extramedullary plasmacytoma [1, 3]. Extramedullary involvement usually occurs in advanced disease. Direct involvement of myeloma causing pleural effusion is rare and occurs in less than one percent of patients [4]. We report a case of myelomatous pleural effusion (MPE) in a patient with known immunoglobulin A (IgA) kappa multiple myeloma that was confirmed by cytology and immunohistochemical (IHC) staining. Informed consent statement was obtained for this study.

## Case presentation

**Figure 1:**
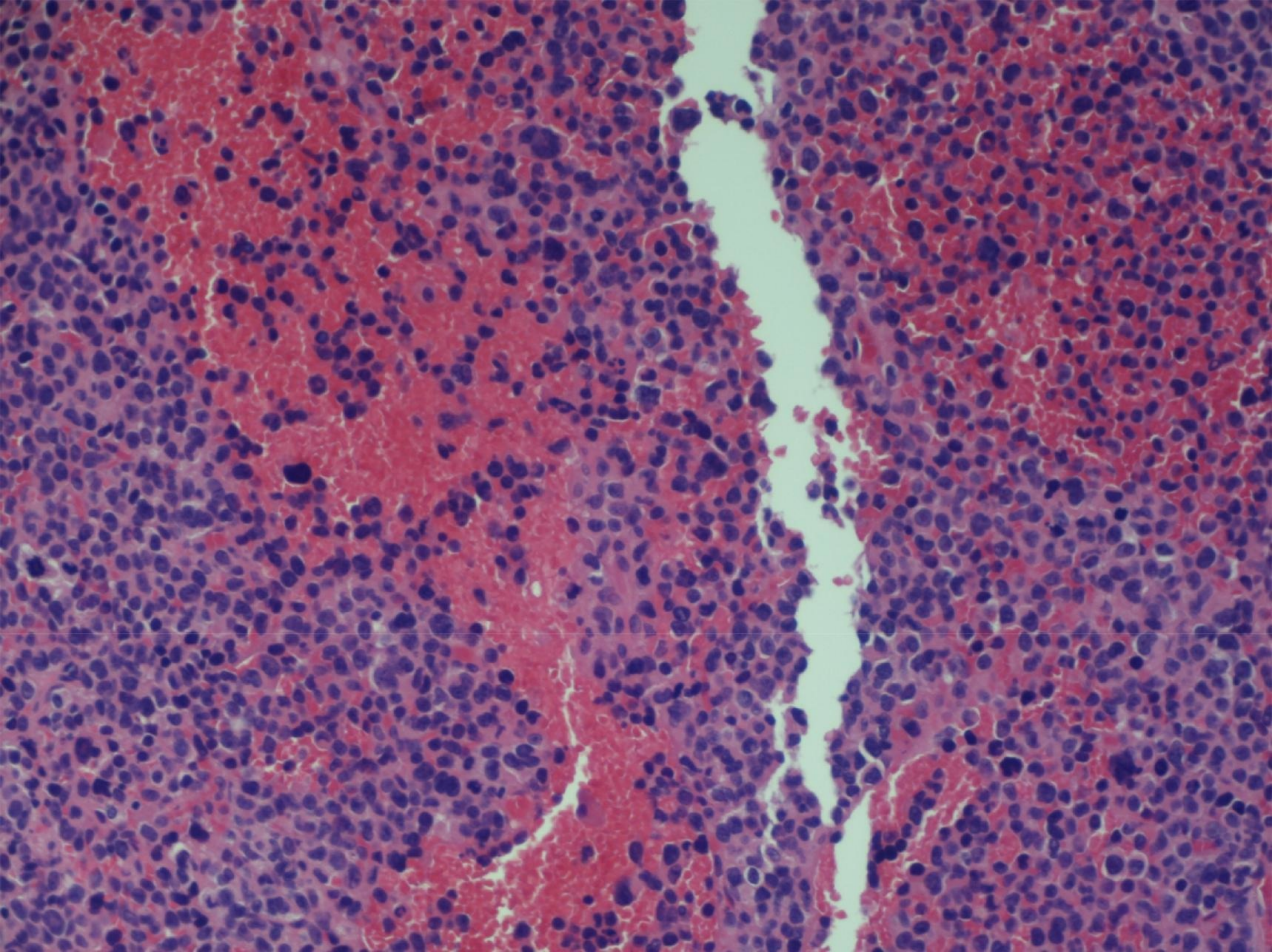
Hypercellular bone marrow with 90% involvement by plasma cells

**Figure 2:**
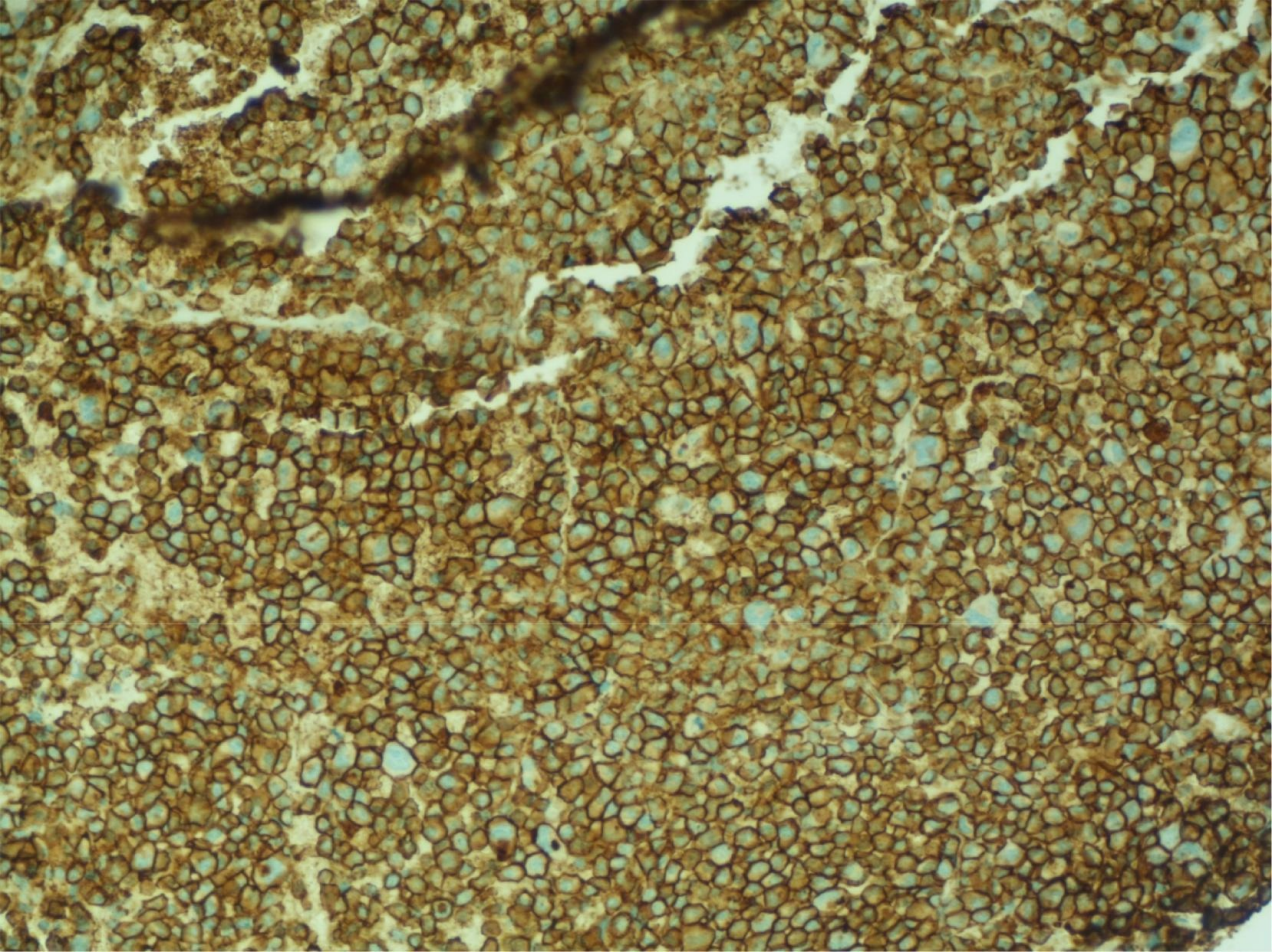
Plasma cells with diffuse kappa light chain positivity by immunohistochemical (IHC)

**Figure 3:**
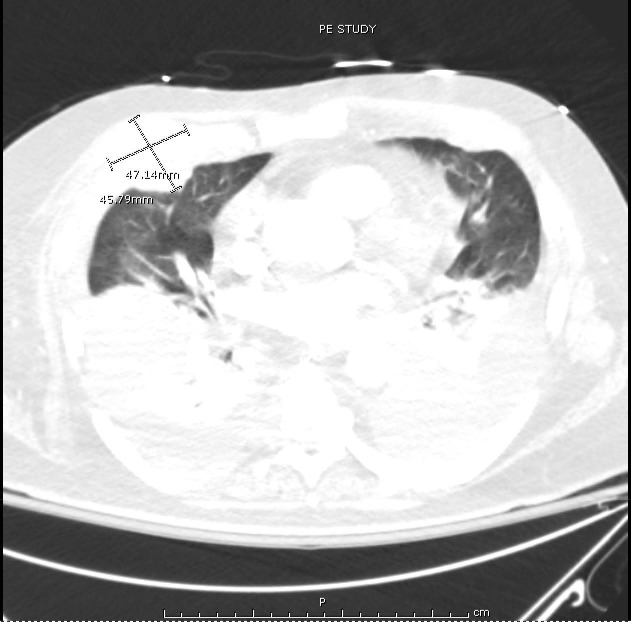
Computed tomography (CT) of chest showing widespread lobulated masses present bilaterally, largest measuring 4.6 x 4.7 cm in the right mid chest with bilateral pleural effusions causing severe compressive atelectasis

**Figure 4:**
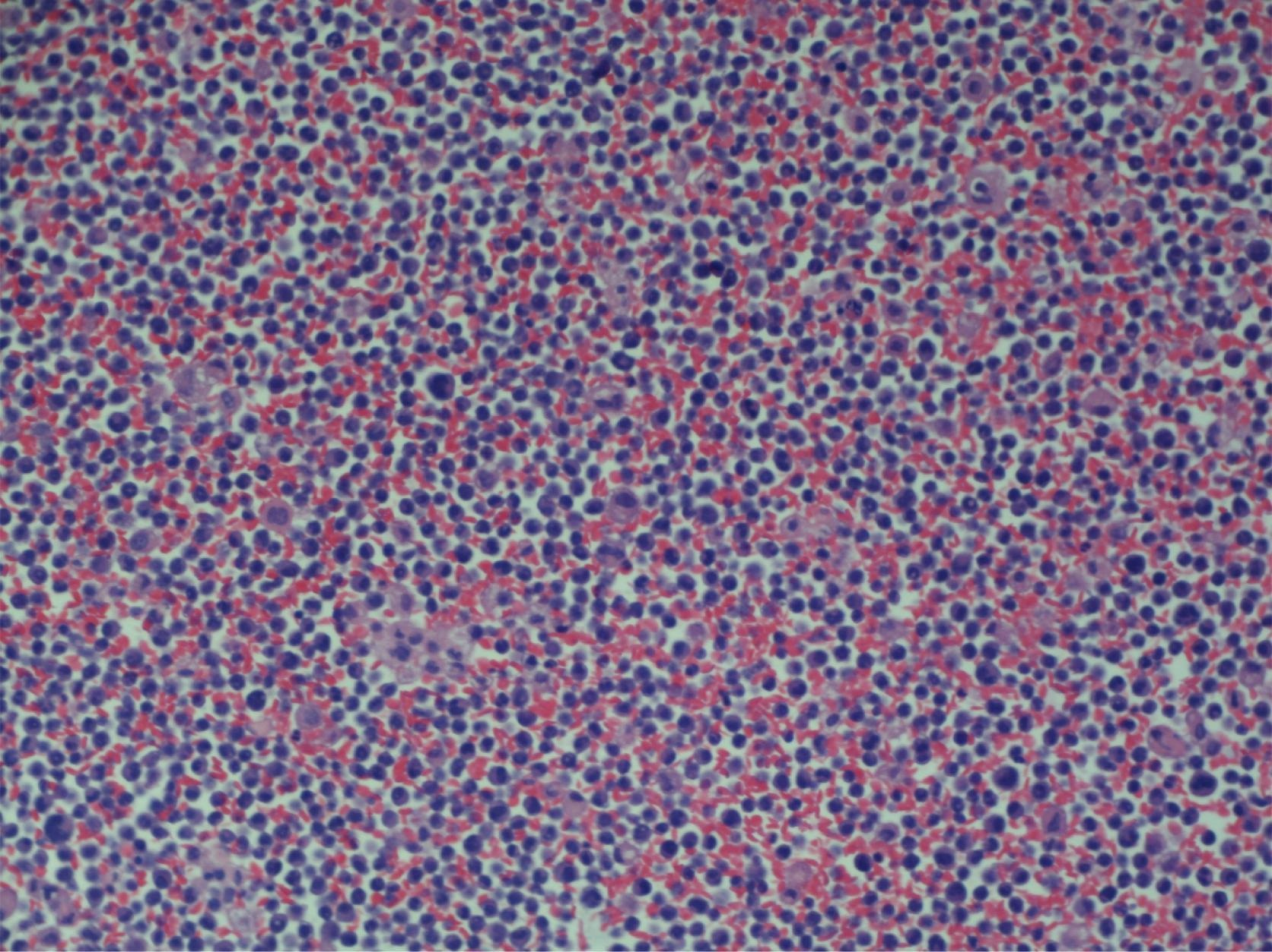
Abundant atypical plasma cells in thoracentesis fluid

**Figure 5:**
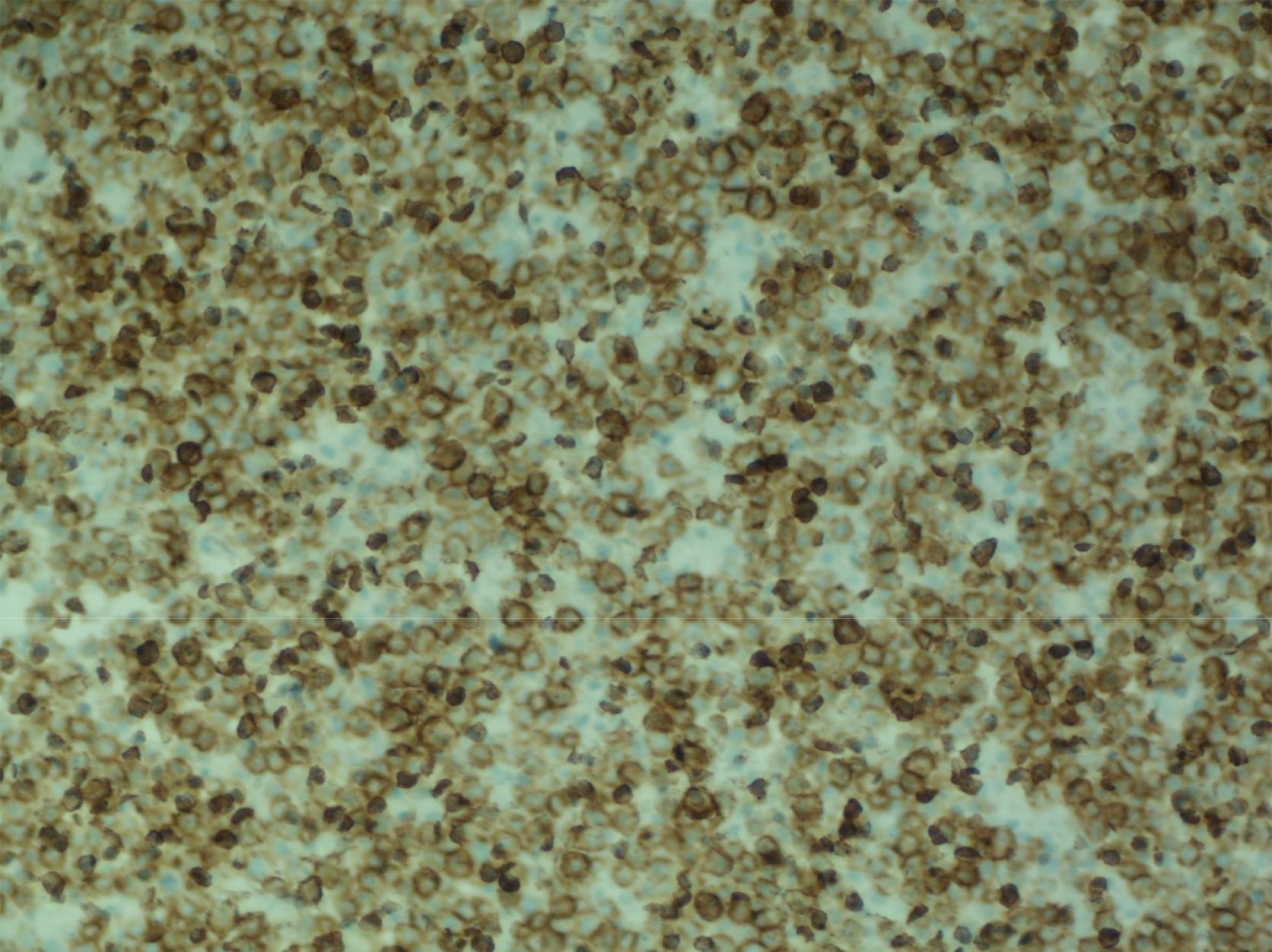
Immunohistochemical (IHC) stain highlighting CD138 positive plasma cells in thoracentesis fluid

A 68-year-old male with stage III IgA kappa multiple myeloma who had completed two cycles of chemotherapy using bortezomib and dexamethasone presented to the hospital for shortness of breath and respiratory distress. His diagnosis of multiple myeloma was made four months prior after he was found to have lytic lesions in the right humerus, left tenth thoracic vertebra, left first lumbar vertebra and right sacrum on imaging performed after a right humeral pathological fracture. The imaging also revealed a large left chest wall mass, thought to be likely from extraosseous involvement by myeloma. Staging workup was completed including skeletal survey and bone marrow biopsy. Bone marrow biopsy showed hypercellularity with 90% involvement by atypical plasma cells which appear as large-sized, binucleated cells with dense chromatin and prominent nucleoli (Figure 1). Syndecan-1 (CD138) and kappa light chain IHC staining showed extensive plasma cell involvement belonging to kappa subtype (Figures 2). The cytogenetic analysis also revealed multiple chromosomal abnormalities compatible with a complex cytogenetic profile of male karyotype with derivative chromosomes 17, 14 and 10. Serum protein electrophoresis detected a monoclonal spike, with immunofixation detecting IgA kappa type. Serum IgA levels were 2519 mg/dL (normal reference range: 66-436 mg/dL); immunoglobulin G (IgG) and immunoglobulin M (IgM) levels were decreased. The patient was determined to have stage III using the revised international staging system (R-ISS). The treatment plan was to start the patient on nine to 12 weeks of bortezomib, lenalidomide and dexamethasone velcade, revlimid, low dose (VRd) regimen followed by re-staging. However, the patient did not receive lenalidomide due to poor performance status and underlying comorbidities. He then completed two cycles of bortezomib and dexamethasone before presenting to the hospital. At the time of presentation to the emergency room (ER), the patient was in respiratory distress and required intubation. On computerized axial tomography (CAT), on the scan of chest with contrast, the patient was noted to have large bilateral pleural effusions with severe compressive atelectasis and in addition to previously seen chest wall mass, there were new widespread pleural and paraspinal metastases (Figure 3). Thoracentesis was performed and upon analysis, fluid was determined to be exudative containing 17,240 white blood cells with 84% being atypical plasmacytoid cells (Figure 4); Thoracentesis fluid lactate dehydrogenase (LDH) was 665 U/L, total protein was 4.5 g/dL, albumin was 1.5 g/dL and potential of hydrogen (pH) was 7.371. Patient’s serum LDH was 475 U/L and total protein was 7.6 g/dL. IHC staining of thoracentesis fluid further revealed CD138, CD31, and kappa light chain positive plasma cells (Figure 5) which was consistent with MPE. Due to patient’s respiratory status and progression of his disease, patient’s family decided to terminally extubate the patient. After extubation patient was placed on comfort measures only (CMO) and he passed away within the next twelve hours.

## Discussion

Pleural effusion and diffuse pulmonary involvement from multiple myeloma are rare with a frequency of only six percent, from all causes of pleural effusions in the setting of multiple myeloma [5]. Most cases of pleural effusions in multiple myeloma are related to associated pathologies such as pulmonary embolism, nephrotic syndrome and heart failure [3-6]. Pleural effusions are directly attributable to multiple myeloma, i.e. occurring from direct plasma cell infiltration and is seen in less than one percent of cases and less than 100 cases have been reported worldwide [3]. The majority of cases have immunoglobulin A (IgA) disease, approximating to 80% of all cases. A likely explanation for the preponderance of cases being caused by the IgA subtype could be due to increased tendency for this subtype to involve extraosseous structures including liver, spleen, and lymph nodes. Though the exact pathogenesis of MPE remains unclear, it is thought to be likely related to direct extension from thoracic skeletal lesions or chest wall plasmacytomas [3]. In the present case, the patient did have direct bilateral chest wall and pleural metastatic involvement as seen on computed tomography (CT) scan, which could explain the etiology of bilateral pleural effusions.

Rodriguez, et al. proposed three potential diagnostic criteria to confirm MPE: (i) demonstration of monoclonal protein in pleural fluid electrophoresis, (ii) detection of atypical plasma cells in pleural fluid and (iii) histological confirmation with a pleural biopsy made at autopsy [7]. This was the first case reported in 1994. Since then, there have been other cases reported, that had not always performed all the aforementioned procedures, including electrophoresis, cytology, and biopsy, to confirm the diagnosis. In addition to aforementioned diagnostics, sometimes, thoracoscopy and bone marrow biopsy have also been performed to diagnose MPE [5]. It is paramount to exclude all other causes of pleural effusion using clinical judgment prior to making a definitive diagnosis of MPE. Thus we postulate that although there have been suggested criteria to attempt and define MPE, they are certainly helpful but not definitive in reaching the diagnosis of MPE. The expertise and ability of the pathologist to make the diagnosis from cytology and IHC is also essential and can prevent unnecessary and sometimes invasive diagnostic testing. In this case, the cytology and IHC staining were sufficient to make the diagnosis of MPE since the patient was already diagnosed with multiple myeloma. If the diagnosis remains unclear after cytological and IHC analysis, additional testing including flow cytometry, protein electrophoresis, and pleural biopsy may be employed to aid in the diagnosis [6-7].

## Conclusions

MPE confers a poor prognosis and is generally seen in advanced diseases. The current case highlights the importance of appropriate diagnostic workup of patients with a history of multiple myeloma presenting with pleural effusion. A similar approach can be employed in identifying MPE without a known diagnosis of multiple myeloma, although a higher index of suspicion, exclusion of other common etiologies and utilization of all available diagnostic modalities might be needed. Since the exact pathogenesis of MPE remains unclear, there is a need to explore predictive and prognostic factors which could further help in the development of better treatment options for myelomas with MPE.
